# Implications of different membrane compartmentalization models in particle-based *in silico* studies

**DOI:** 10.1098/rsos.221177

**Published:** 2023-07-05

**Authors:** Philipp Henning, Till Köster, Fiete Haack, Kevin Burrage, Adelinde M. Uhrmacher

**Affiliations:** ^1^ Institute for Visual and Analytic Computing, University of Rostock, Rostock, Germany; ^2^ School of Mathematical Sciences, Queensland University of Technology, Brisbane, Australia; ^3^ Visiting Professor, Department of Computer Science, University of Oxford, Oxford, UK

**Keywords:** particle-based simulation, membrane, restricted diffusion

## Abstract

Studying membrane dynamics is important to understand the cellular response to environmental stimuli. A decisive spatial characteristic of the plasma membrane is its compartmental structure created by the actin-based membrane-skeleton (fences) and anchored transmembrane proteins (pickets). Particle-based reaction–diffusion simulation of the membrane offers a suitable temporal and spatial resolution to analyse its spatially heterogeneous and stochastic dynamics. Fences have been modelled via hop probabilities, potentials or explicit picket fences. Our study analyses the different approaches’ constraints and their impact on simulation results and performance. Each of the methods comes with its own constraints; the picket fences require small timesteps, potential fences might induce a bias in diffusion in crowded systems, and probabilistic fences, in addition to carefully scaling the probability with the timesteps, induce higher computational costs for each propagation step.

## 1. Introduction

The plasma membrane constitutes a complex biochemical and mechanical structure that mediates the interaction of cells with the external environment and thus plays a key role in the response of cells to external stimuli. For intracellular and intercellular signalling, the lateral organization and mobility that controls the interaction between membrane-associated components (such as receptor dimerization or signalosome formation) are as important as the interaction between the membrane and the extracellular environment (e.g. ligand/receptor binding) [1,2]. In the past, the former aspect has long been underestimated and neglected [3–5]. This has also been criticized in systems biology due to the role of the membrane’s spatial organization for quantitatively understanding signal transduction pathways [6].

For many cell biological and biochemical studies, it is crucial to understand the mechanisms and organization of the membrane, especially if the organization of the membrane is changed on purpose, as is done, for example, by applying an external electric field for medical treatment [7,8]. The exact mechanisms behind the cellular response to external electrical fields are still unknown [9]. Part of a potential explanation is the drift of proteins and micro-domains in the membrane due to the external electric field [10–12].

Within the last decade(s), the development of advanced microscopic techniques has allowed for a more detailed characterization of the complex and dynamic membrane composition and structure of the membrane, and membrane-based reactions [13,14]. Various experiments, *in vitro* as well as in model membranes, have been conducted to further understand the interplay between membrane-linked processes and the structuring of the membrane [15]. These experiments are increasingly complemented by *in silico* studies.

Thereby, one commonly used approach is molecular dynamic (MD) simulations that offer a high degree of detail. These allow the simulation of a variety of processes in the membrane, such as the formation of different lipid domains [16], protein oligomerization [17] and many more. The level of detail of these models varies from all-atom models that take all atoms of the proteins, lipids and solvent into account to implicit models that reduce the protein to a qualitative few-bead model and the lipids and solvent to a mean-field approach [18]. Despite these reductions in complexity, the MD models remain computationally expensive and are therefore limited to small systems and short timescales. All-atom models are capable of simulating bilayers of approximately 250 lipids for several hundred nanoseconds. By reducing the proteins and lipids to a few beads per molecule and the solvent to a mean-field, systems with several million particles (length scale less than 100 nm) can be simulated up to the millisecond range. Even these reduced models are hardly capable to capture the membrane compartmentalization that takes place on the scale of several hundred nanometres and times in the range of up to approximately 500 ns [19]. Despite the high computational cost of MD simulations, Koldsø *et al.* [20] were able to simulate the reduction of the mobility of lipids and proteins in a coarse-grained MD simulation containing fixed proteins (pickets). The membrane was composed of 2.69 million particles resulting in a size of 146 × 146 nm and was simulated for 10 μs. Despite this achievement, Enkavi *et al.* [17] stated ‘The scales associated with cytoskeleton-induced compartmentalization are large; therefore also lateral diffusion should be considered over time and spatial scales that are far beyond the molecular scales. A question arises whether highly coarse-grained simulation approaches would be more meaningful to consider lateral diffusion under these conditions compared to molecular simulation models.’

One class of these highly coarse-grained simulation approaches are particle-based reaction–diffusion simulations [21]. This approach only simulates particles of interest, abstracted as spheres, that diffuse according to the overdamped Langevin equation. Despite its name, the method also samples the interaction of pairs of particles and can satisfy both unimolecular and bimolecular reactions. In systems biology, this is often called the mesoscale level in spatial modelling [22] and is supported by a variety of tools [23,24], such as ReaDDy [25], SpringSaLaD [26], Smoldyn [27], NERDSS [28] and Spatiocyte [29].

Such an approach was used to investigate membrane dynamics such as lipid rafts motion due to electrical fields [12] and the diffusion of individual lipids [30] or proteins [19] in the cell membrane. The low degree of detail makes the simulation less computing-intensive. It allows the use of time intervals in the range of seconds and regions with edge lengths up to several micrometres, which is needed in order to investigate the effect of the actin skeleton on the diffusion of particles and the potential formation of signalosomes via clustering in the membrane.

The way the fences are modelled in these particle-based reaction–diffusion simulations varies. For example, Andrade *et al.* [30] and colleagues [31] use infinite thin barriers that can be crossed with a certain hop probability. While these two studies used the approach in continuous space, this type of restriction can also be implemented in lattice-based simulations [32]. Another approach to model the fences is by fixed particles (actin-bound proteins), which was done in lattice-based simulations [33,34] as well as one of the few coarse-grained MD simulations that consider fences [20]. A third way to create fenced regions in a simulation is by explicitly modelling the filaments’ electrostatic effects as potentials [35].

**Figure 1.**
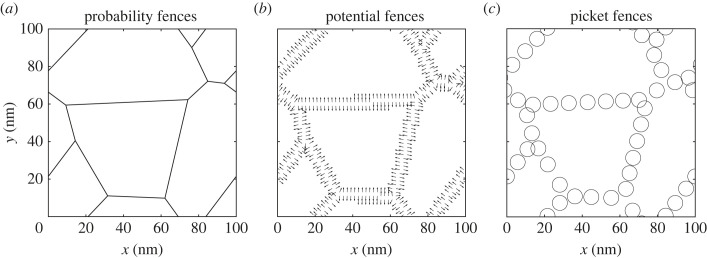
(*a*) The probabilistic fences, infinite thin barriers that can be crossed with a predefined probability (*P*_Hop_), (*b*) the potential fences repulse particles in their proximity (closer than *x*_0_) with a force of *k*_fence_ as indicated by the arrows showing the direction of the force and (*c*) the picket fences, that consist of immobile particles that repulse other particles and thereby create excluded areas. All fences shown use a characteristic length (see §2.1) of *L* = 50 nm and are based on a periodic Voronoi diagram.

Since it has been shown that the chosen particle-based approach has an impact on the quantitative results when simulating biochemical reactions [21], the question is whether and how different approaches to model the fences might influence quantitative results (and computational cost). In the following, we will hone in on the question of how to model fences in particle-based simulations of the membrane and study the impact of the chosen approach. Therefore, we will focus on three different ways to model fences that have been applied in earlier studies as mentioned above, i.e. probability fences, potential fences, and picket fences (figure 1) and discuss the differences between these three approaches to model fences and compare the computational cost of the simulations.

## 2. Material and methods

In the following paragraphs, we will describe the central concepts of our particle-based simulator.

### 2.1. Propagation model and implementation

The diffusion of particles is simulated by an overdamped Langevin propagator (equation (2.1)), as it is also used in various other particle-based simulators, see [25–28,36] in a similar form. The overdamped propagator assumes that the momentum a particle carries due to the diffusion process vanishes within a single timestep. Since the simulator is specifically designed to simulate membranes, the movement of particles is restricted to a two-dimensional plane with periodic boundary conditions. In our realization of the propagator, one term takes the diffusion process into account, and the other terms describe the forces that act on a particle. The different types of forces are those due to particle–particle, particle–fence interaction and of globally applied forces leading to a drift of the particle. The propagator for the change of position $x_{i}$ of particle *i* is defined as:

2.1 $$\Delta x_{i}=\delta_{\mathrm{crossing}-\mathrm{test}}\left(\underset{\mathrm{diffusion}}{\underbrace{\sqrt{2D_{0}\cdot dt}\;\xi }}+\underset{\mathrm{forces}}{\underbrace{\frac{dt}{\gamma }\left(\overset{\mathrm{particle}-\mathrm{particle}\;\mathrm{interaction}}{\overbrace{\sum_{\;j\neq i}\frac{\left|F_{p_{i}-p_{j}}(\Delta x_{i,j})\right|}{\left|\Delta x_{i,j}\right|}\mathrm{if}\Delta x_{i,j}}}+\overset{\mathrm{fence}\;\mathrm{potentials}}{\overbrace{\sum_{k}F_{p_{i}-f_{k}}}}+\gamma v_{\mathrm{drift}}\right)}}\right).$$

The propagator’s first term describes the diffusion of the particle as the mean random effect of the surrounding system with two normally distributed random numbers (ξ) for its displacement in the *x*-direction and *y*-direction.

Various forces act on the particles. Forces between particles push those particles apart with a force expression specified in the model. We use a simple linear expression based on the overlap. This force is identical for interactions between two freely moving particles and between a free particle and an immobile picket fence particle. Both types of particles are accounted for in the sum over *j*.

In the case of a potential approach to model fences, a term for this force acting on the particles is added (*fence potentials*). The magnitude of the force (*k*_fence_) is determined by the modeller. Each fence corresponds to one force vector on each particle based on the chosen potential shape and the distance from the fence line. The magnitude of the force is determined only by the strongest force vector (which is the nearest neighbour) to avoid very high forces in corners (figure 1). The direction is determined by the vector sum of all force vectors. We use a triangular-shaped potential for the force. Note that the used potential does not represent a single physical effect such as electrostatic forces [35] but is an abstraction of all effects that restrict the movement of particles (pickets, actin filaments, electrostatic repulsion), similar to the hop approach representing all restricting effects on particle diffusion by one aggregate random value. The drift force is a global force that leads to a constant drift in particle movement.

Finally, if the $\Delta x_{i}$ would lead to a particle crossing a fence, the crossing test is performed. A uniformly distributed random value is generated in the interval [0, 1] and compared with the hop probability (*P*_Hop_), accordingly the prefactor *δ*_crossing−*test*_ is set either to zero or one. If the test fails, the particle remains at its position. A discussion of other methods to handle the failed crossing of a fence can be found in the electronic supplementary material.

We have implemented our test simulator in the Rust programming language [37]. This allows for fast native code execution, and its type system provides strong static guarantees referring to memory and thread safety during compilation. The simulator and the code to generate the plots and parameter fitting within this paper are available at https://doi.org/10.17605/OSF.IO/MHX7D [38]. Each simulation run is specified via a configuration file to ensure a separation between the model and the simulator. This file contains the parameters as outlined in table 1 as well as some execution-specific parameters, including the number of replications and how to handle observables.

**Table 1.** Overview of the model parameters and their typical values.

parameter	symbol	typical values
system		
number of particles		1–2000
radius of particles	*r*	0.4–2 nm [39,40]
diffusion coefficient of free particles	*D*_0_	0.8 μm^2^ s^−1^ [30]
temperature	*T*	300 K
drag coefficient of the membrane and aqueous phase	*γ*	*T* · *k*_Boltzmann_/*D*_0_
force between overlapping particles (magnitude)	$\left|F_{p_{i}-p_{j}}(\Delta x_{i,j})\right|$	(Δ*x*_*i*,*j*_ − *r*_*i*_ − *r*_*j*_) · 0.075 to 0.15 rm kg s^−1^
drift velocity of particles	$v_{\mathrm{drift}}$	≤1 μm s^−1^ [41,42]
fences		
characteristic length	*L*	50–200 nm [19]
energy of the potential fence	*k*_fence_	0–0.04 aJ
width of potential	*x*_0_	2.5–5.0 nm [43]
force of a fence on a particle	$F_{p_{i}-f_{k}}(d=\mathrm{distance})$	*k*_fence_/*x*_0_ if *d* < *x*_0_
hop probability across a fence	*P*_Hop_	0–100%
radius of pickets	*R*	2–4 nm [44–46]
mean distance between pickets	*l*_free_	0.4–1.2 nm
simulation		
timestep	d*t*	2–2000 ns
simulated duration		<20 s

The actin-mesh is modelled as a (periodic) Voronoi diagram (see also figure 1), a method that has been shown to work well in representing biological structures [47]. Independently of the fence model, all fences are described by the characteristic length *L* of the Voronoi mesh that is defined as the mean square root of fenced regions. The periodic Voronoi diagram is generated by placing *x* ∗ *y*/*L*^2^ randomly distributed points in the simulation area. To enforce a periodic mesh, the points are copied and placed around the simulation area in a Moore neighbourhood manner. A Voronoi diagram is generated from all these points, and the characteristic length *L*_mesh_ is calculated. If *L*_mesh_ differs for more than 2% from the desired *L*, the process is repeated with either one point more or one point less.

### 2.2. Simulation parameters and output

In this section, we will describe the parameters used in most of the simulations. If different values are used, it is stated in the corresponding section. Most simulations use a particle radius of 0.4 nm. This radius is in the range of a lipid radii [38,40]. It is a relatively small particle in the membrane, and especially for models that include reactions, larger particles such as proteins are more of interest. We decided to use a small lipid for most tests as it is more challenging to sample interactions correctly for smaller particles since we want to test the fence models under these challenging conditions. For the same reason, a small characteristic length *L* of 50 nm [19] is mainly used. Since this *L* is relatively small and we are mainly using periodic boundaries, our simulation region is 100 × 100 nm large. If a larger *L* is used, the simulation region should be enlarged to find a Voronoi mesh with the corresponding *L*. The picket fences are built from pickets with radius *R* = 4 nm and a mean free space of 0.8 nm between adjacent pickets. The potential fences are defined by a triangular potential with an energy *k*_fence_ that primarily affects the restriction and a width *x*_0_ = 2.5 nm. Here, *x*_0_ is roughly the radius of an actin filament [43]. For the probabilistic fences, the restrictions are defined by the hop probability *P*_Hop_. The diffusion coefficient *D*_0_ of 0.8 μm^2^ s^−1^ is taken from measurements of di-palmitoyl-phosphoethanolamine (DPPE) in a compartmentalized membrane [30] of NRK and IA32 cells. For the experiments involving a drift, a velocity of *v*_drift_ = 1 μm s^−1^ is used. This drift velocity is in the range of ones reported in model membranes [41,42].

The outputs of the simulations are the trajectories of each individual particle and the mean squared displacement (MSD$(t)=\sum_{i=1}^{N}{\left(r_{i}(t+\tau )-r_{i}(\tau )\right)}^{2}$) of all *N* particles in the system every 20 μs. From the MSD, the diffusion coefficient can be calculated (*D*(*t*) = MSD(*t*)/(2 · *n* · *t*)), where *n* is the dimension. In single-particle tracking studies, it was observed that the calculated diffusion coefficient differs for different time resolutions (*t*) [19,48]. For a high video rate, the free (microscopic) diffusion is observed on a short time scale and the slower restricted (macroscopic) diffusion on a longer time scale.

An alternative method to observe particles is the fluorescence correlation spectroscopy (FCS) [49]. Here, no individual particles but the fluorescence signal are observed in the focal area of the microscope. From the auto-correlation function of the fluorescence signal, the diffusion coefficient can be calculated [49]. Andrade *et al.* [30] observed that for a larger focal area, the diffusion appears to be slower due to the restriction of fences (macroscopic *D*) in the focal area. For focal areas small compared with *L*, the microscopic *D* is observed. With the help of particle-based simulations fitted to their wet-laboratory results, they gain further information about their system, namely, the characteristic length *L*, the microscopic diffusion coefficient *D* and the hop probability of a particle, information that could not be derived from the FCS measurement without simulations.

We use the first method to calculate the diffusion coefficient for different time resolutions. This can be done directly from the output. Analysing the diffusion in the same way as done in FCS studies would also be possible. However, it requires additional steps. The fluorescence signals from different focal areas need to be calculated from the trajectories. In the same way, as done with wet-laboratory data, the diffusion coefficient can be calculated from the auto-correlation of the signals [49].

## 3. Results

Our study comprises the following steps. First, we compare the simulation results with an exact solution of a simple benchmark to check the three fence models for correctness (§3.1). In addition to the correctness, the simulation’s performance is decisive when selecting a method. Therefore, we investigated both computational costs per timestep (§3.2) and timestep convergence (§3.3) in single-particle and many-particle systems. To elucidate the differences and similarities between the fence models, we fit their parameters to yield similar results (§3.4). One key difference between the fence models is their response to particle density (§3.5). Another issue is how the different fence models cope with external forces on the particles. Therefore, in §3.6, a drift component is added, and the simulation results of the three fence models are compared.

### 3.1. Comparison with the exact solution

**Figure 2.**
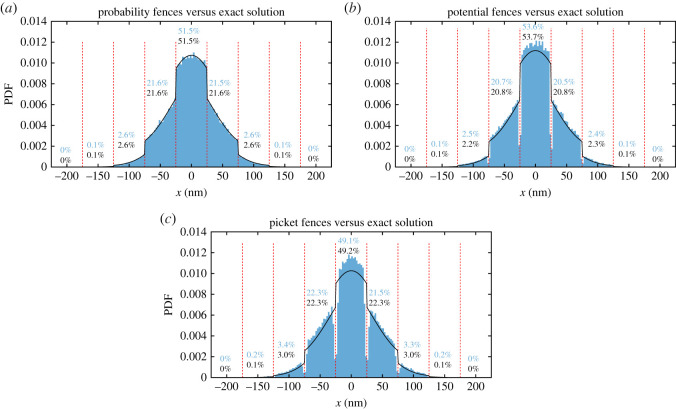
Simulated particle distribution in the *x*-direction (blue) and exact distribution (black) according to [50] for (*a*) probabilistic fences, (*b*) potential fences and (*c*) picket fences after *t* = 1.58 ms. The dashed red lines show the barriers. All particles start at *x* = 0 nm with 100 000 replications. The numbers show the probability of a particle being located in the respective compartment for the simulation (blue) and exact (black) solution.

To ensure the soundness of our particle-based simulation, we first run a simple test case of a single particle that can diffuse freely in the *y*-direction but is restricted by a barrier every 50 nm in the *x*-direction. For this case of equally spaced, partially permeable barriers in an infinite domain and the initial distribution of a delta distribution, the exact analytical solution is known [50]. The simulation results were obtained by placing a single particle in the centre between two barriers and letting it diffuse for 1.58 ms with a sufficiently small timestep (see §3.3). The simulation box has a length of 2000 nm in the *x*-direction. This length is much larger than the distance travelled by a particle during the simulation. The permeability of the barriers in the exact solution is chosen, with a least-square method, so that the probability density function (PDF) between the barriers matches as closely as possible to the one obtained from the particle-based simulation. For the probabilistic fences (figure 2*a*), the exact particle distribution matches closely with the distribution from our particle-based simulation. In the case of the potential and picket fence (figure 2*b*,*c*), the simulated distribution differs slightly from the exact solution. This is because both fences have a finite thickness, while the exact solution assumes an infinite thin barrier. Macroscopically, the probability of a particle being located in a compartment matches the exact solution. However, microscopically, due to the repulsion from the barrier, fewer particles are located close to the barrier. Nevertheless, the general shape of the simulated distributions matches those of the exact solution. We note that there are other possibilities for an analytical model based around the Narrow Escape problem [51]. In this setting, particles can diffuse through a narrow absorbing window in a domain with otherwise reflecting boundaries. The mean first passage time for exit can be computed analytically in terms of a mixed Neumann–Dirichlet boundary value problem. It is possible to extend this to the case where there are a number (usually the same size) of well-separated structures. Furthermore, effective diffusion on a membrane with obstacles can be represented as a Narrow Escape problem by, for example, considering diffusion in a plane populated with reflecting circles of the same size acting as obstacles [51]. More recently, Meiser *et al.* [52] have tested the Narrow Escape theory on a model system of micro-patterned lipid bilayers from experimental and stochastic simulation perspectives.

### 3.2. Performance

As discussed in the Introduction, the high computational cost of detailed MD simulations makes it challenging to study the effect of actin skeleton-induced compartmentalization on lateral diffusion. The computational costs result from the large number of particles and their interactions in the system and the small timestep, typically in the range of femtoseconds.

By using a particle-based reaction–diffusion method, our simulations are significantly less detailed, and the number of particles is reduced by several orders of magnitude. However, the computational cost of a simulation might depend on the chosen fence model. Therefore, we take a look at the performance of the three fence models and some details of the simulator in this section.

To achieve the necessary performance, we need to exploit spatial locality within the simulation. To do this, we use spatial lookup tables. We have both a static lookup table (for the fence information) and a dynamic lookup table (for the positions of the mobile particles). These lookup tables are used to find particles or lattice elements near each particle and avoid unnecessary computations for fences or particles far away. Namely, we subdivide the space into voxels. A list of particles/fences interacting in that area is stored for each voxel. The voxels are stored in a two-dimensional array. For a very small number of particles (in our case, less than 16), a brute force approach (testing each particle–particle pair independent of location and without ever creating a lookup table) was the fastest as it saves overheads when only a few particles are considered. However, it scales badly for a larger number of particles.

To better understand the performance characteristics of the implementations, we conducted a small performance study. We tested the three implemented realizations of fences by varying the numbers of (moving) particles within two membranes of different sizes.

— The numbers of particles were varied between 1 and 100 000.
— Two membranes of different sizes were considered (small and large corresponding to 100 × 100 nm and 2000 × 2000 nm, with the characteristic length *L* set to 50 nm).
— Executing the three models of fences (probabilistic, potential, pickets) and a free (no fence) system as a benchmark.

**Figure 3.**
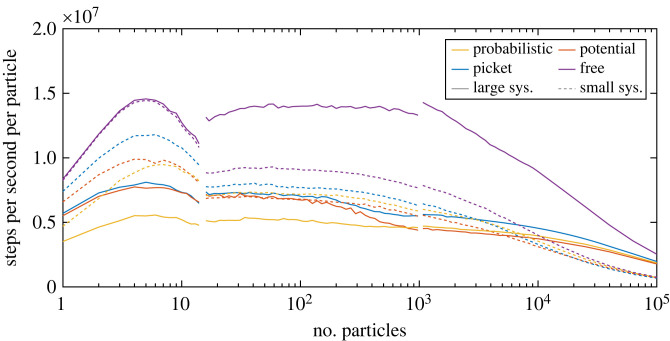
An overview of the throughput of the different methods. Compared are a varying number of particles within two different large regions of simulations of different large membrane regions (2000 × 2000 nm for large and 100 × 100 nm for small) and different numbers of particles. The discontinuities in the lines mark the algorithmic configuration shift points. Here, we change algorithmic paramet rization to suit the respective regime better.

The exact same fence geometry (i.e. Voronoi diagram) is used for the three different models. The density of the fences is also the same across both system/membrane region variants. Therefore, the absolute number of fences is larger for the larger region. For the same number of particles, the particle density is lower when considering a larger region. The results are shown in figure 3.

The different colours represent the different models. The dashed lines are for the smaller region. On the *x*-axis, we have the number of particles. On the *y*-axis, the performance metric is shown, which is the number of particles updated per second of wallclock time. The measurements were carried out on a single thread of a desktop workstation with an Intel Xeon E5-1630 CPU.

We can observe several trends in this data. Initially, performance per particle increases with an increasing number of particles. This is expected as operations done once per step amortize when increasing the number of particles. After about 10 particles, we can see a strong decline in performance for the small system but not as pronounced for the large system. This is because, in the small region, collisions (or, more generally, checking for collisions for nearby particles) occur more frequently. For small particle numbers (fewer than 16), we also use an adapted algorithm (as described above) that is more optimized for small system sizes. Furthermore, smaller copy numbers are also more cache-friendly.

We can also observe the performance of the different methods of implementing fences. The picket fence displays the best performance. This is because the interaction is local (only when pickets and particles touch) and cheap to compute (because a linear force is used). The potential-based approach is slower as potentials have a longer range (less locality to exploit). The probabilistic fence is the computationally most expensive. While the interaction is equally local as the pickets, it requires a random number as well as more complicated geometrical calculations for line intersections.

For the small membrane region, the overhead due to using fences compared with free particles is relatively small, as the total number of barriers (not the density) in the system is low, and particle–particle interaction dominates runtime. For a larger region, the lookup becomes more expensive, and the difference between the free system and fences increases.

Since the number of particles simulated in this case study is relatively small, not all avenues to tune performance for larger numbers of particles have been explored. We expect performance could be improved for dense systems.

### 3.3. The importance of selecting suitable timesteps

Additionally, to use a fence model that is executed quickly, the performance can be increased by using a large timestep, reducing the overall number of steps needed in a simulation run. This raises the question of selecting the right timestep to balance the need between efficiency and accuracy. Therefore, we will analyse how sensitive the methods are to varying timesteps.

**Figure 4.**
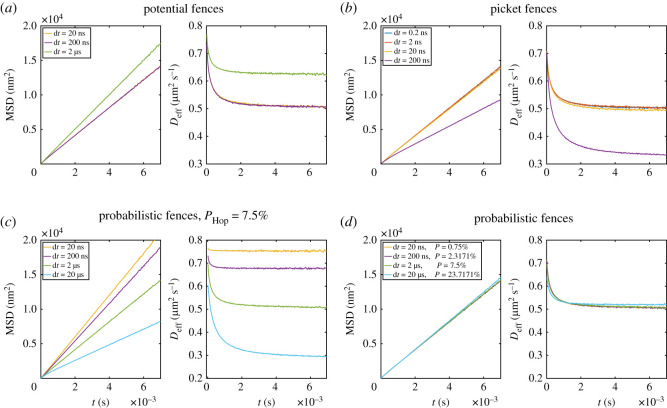
MSD and effective diffusion coefficient over time for (*a*) the potential-based fence, (*b*) for picket fences, (*c*) probabilistic fences with the same $P_{\mathrm{Hop}}=7.5\%$ for all tested timesteps and (*d*) for a scaled ${\tilde{P}}_{\mathrm{Hop}}$ of $0.1677\%/\sqrt{ns}$ for different timesteps. The value of d*t*, as well as the unscaled *P*_Hop_ are shown in the legend.

As simulation parameters, we use the ones specified in §2.2. The Voronoi diagram used is the same as shown in figure 1. Runs with the potential-based fences use an energy of *k*_fence_ = 0.0141 aJ that restrict the movement as strongly as the picket fences. The results are averaged over 512 replications with a single particle per run. For the potential-based fences, the MSD and *D*_eff_ are the same for a timestep of 20 and 200 ns (figure 4*a*). At the timestep of 2 μs, the MSD and *D*_eff_ differ clearly from the results at the smaller timesteps. Consequently, a timestep of 200 ns is suitable to simulate our parameter configuration. For the picket method, a much smaller timestep of 2 ns (figure 4*b*) needs to be used for the chosen model parameters. Even though the error at d*t* = 20 ns is small, it can be made larger if some parameters are changed, such as the diffusion coefficient or the particle–particle interaction term, and the system becomes more sensitive to the timestep.

In the case of potential-based fences and a large timestep of 20 μs (see electronic supplementary material), we observe an effective diffusion coefficient greater than *D*_0_, showcasing the importance of an appropriate timestep. This is probably due to the repulsive force acting unrealistically long on the particles due to the large timestep. In the case of the picket fences, a too big timestep leads to a stronger restriction on the movement. In contrast to the potential fences, which the moving particles can cross, the pickets create excluded regions with free spaces. As a result, the particle–picket potential is much steeper than for the potential fences, and therefore a smaller timestep is needed to sample the interaction correctly.

In general, the timestep should always be checked for a specific model. Our results/timesteps are only valid for our chosen parameters and cannot be assigned to other models. Nevertheless, they show the range in which one would expect a suitable timestep, and more general trends, such as the need for a smaller timestep for the picket fences, can be transferred to other models.

*Time-dependent hop probability.* In the case of the probabilistic fences, performed with *P*_Hop_ of 7.5%, the results differ significantly for different timesteps as shown in figure 4*c*. The divergence of MSD and *D*_eff_ for different timesteps is not due to a numerical error of the propagator but due to the timestep dependency of *P*_Hop_. This dependency was reported before by Niehaus *et al.* [32]. They observed that the macroscopic diffusion coefficient is larger for smaller timesteps in continuous space methods or smaller lattice constants in lattice-based methods. The same need for scaling *P* with the timestep is discussed in [51,53] for partially absorbing boundaries. To explain why a fixed hop probability will restrict a particle more for a bigger timestep (figure 4*c*), we can imagine a particle that does one step with the timestep d*t*′. The particle has the chance to cross the fence once during this time. If the same particle is propagated with the timestep d*t*′/10, the particle can cross the fence up to 10 times in the same time interval, making it more likely for a particle to cross the fence for a smaller timestep. This effect is partially remedied by the smaller displacement of particles for a smaller timestep, making it less likely to collide with a fence. However, since the displacement scales with the square root of the timestep (using an overdamped Langevin propagator, see equation (2.1)), the sum of both effects results in the scaling of the hop probability with the square root of the timestep. For the scaled hop probability ${\tilde{P}}_{\mathrm{Hop}}=P_{\mathrm{Hop}}/\sqrt{dt}$ the restriction becomes timestep independent for a d*t* up to 2000 ns, as shown in figure 4*d*. In our system, we observed a less restricting effect of the fences for a d*t* larger than 2000 ns. Here, the displacement within a single step is so large that particles can cross more than one fence within a single timestep. This error appears particularly often for small characteristic lengths.

**Figure 5.**
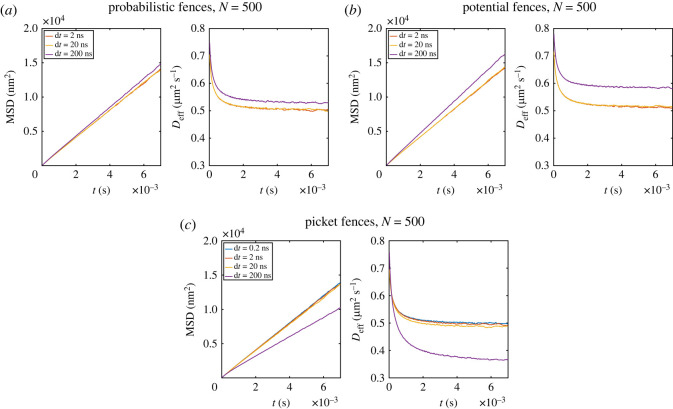
MSD and effective diffusion coefficient over time for (*a*) probability fences, (*b*) potential fences and (*c*) picket fences.

*Timestep dependency for many particles.* Since the simulator also offers the possibility to simulate many particles with interactions, we also checked the timestep for these cases, which are especially of interest for simulations that include bimolecular reactions. For many-particle systems, the critical factor becomes the particle–particle interaction. Hence, we expect a timestep in a similar range as for the picket method. The simulation parameters were the same as described in §2.2 with 500 particles in each run and eight replications. As shown in figure 5, the probabilistic fence and the potential fence are working with a timestep of 20 ns, while the picket fence needs a timestep of 2 ns, as in the simulations with a single particle.

### 3.4. Relating the different fence models

As described in §2.2, each fence model has some specific parameters that determine the restricting effect of the fences.

To understand the relation between the different fence models, we simulated a system with a single, diffusing particle using the probabilistic fence model with *P*_Hop_ between 0.01% and 100% at d*t* = 2000 ns, *D*_0_ = 0.8 μm^2^ s^−1^, and a *L* enhanced to 80 nm. The simulation area is enlarged to 1000 × 1000 nm compared with the simulations done before. The larger simulation region increases the number of possible characteristic lengths achieved in the regions to *L* > 100 nm. To find the values of *k*_fence_, *L* and *D*_0_ that produce the same diffusion behaviour for a single particle (the same *D*_eff_) as the probabilistic fences do by using potential fences, we use an approximate Bayesian computation [54,55] method, namely the tool pyABC [56]. As an output, pyABC gives the posterior distribution over the parameters. We get the most likely value for each tested parameter and its 90% confidence interval from this distribution.

**Figure 6.**
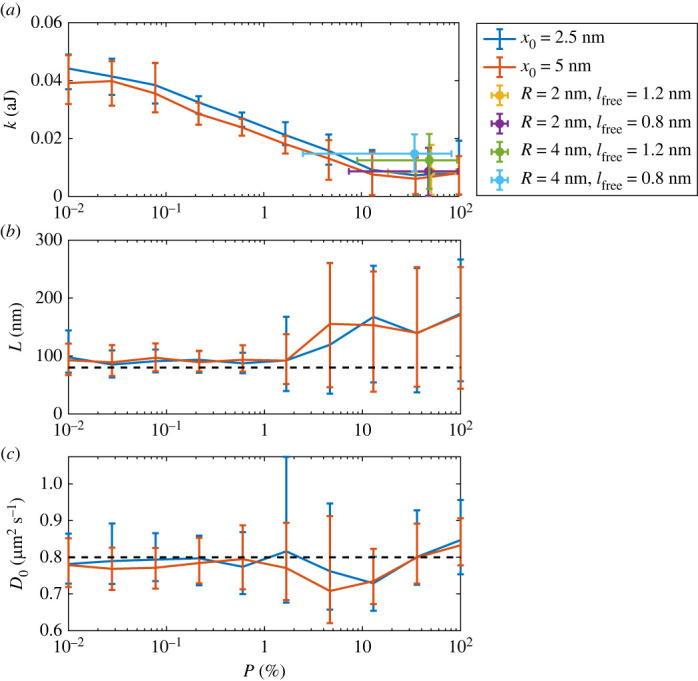
Values of (*a*) *k*_fence_, (*b*) *L* and (*c*) *D*_0_ of the potential fence that gives the closest fit to the *D*_eff_ generated with the probabilistic fence. (*a*) additionally shows the *k*_fence_ and *P*_Hop_ that gives the same *D*_eff_ as picket fences with *R* = 2 nm or *R* = 4 nm and *l*_free_ = 0.8 nm or *l*_free_ = 1.2 nm. The error bars indicate the 90% confidence interval of the posterior distribution and the dashed lines in (*b*) and (*c*) mark the values of *L* = 80 nm and *D*_0_ = 0.8 μm^2^ s^−1^ as chosen in the probabilistic fences.

*k*_fence_, *L* and *D*_0_ were chosen as parameters to test if the broadness of the potential affects the characteristic length of the actin skeleton or if the additional force from the potential fences affects the diffusion. Figure 6 shows the relations that resulted in the fits for two different potential widths (2.5 and 5.0 nm). Additionally, several picket fences configurations (*L* = 80 nm, *D*_0_ = 0.8 μm^2^ s^−1^) were simulated, and characteristic parameters of the potential fences (*k*_fence_, *L* and *D*_0_) and probabilistic fences (*P*_Hop_, *L* and *D*_0_) were fitted to generate the same *D*_eff_. Figure 6 shows the relation between *P*_Hop_ and *k*_fence_ in (*a*), the best *L* to fit probabilistic fence results with the potential fence model in (*b*), and the best *D*_0_ to fit the probabilistic fence results with the potential fence model in (*c*). The potential energy (*k*_fence_) of the fence scales linearly with the logarithm of the hop probability (*P*_Hop_) (figure 6*a*). For the broader potential fence, the energy needed to get the same restriction effect is lower than for the more narrow potential fence. For the fitted characteristic length, we observe that the potential method uses a slightly larger *L*. This can be explained by the different sizes of regions of free diffusion. Probabilistic fences allow particles to move freely in the whole fenced region, while the movement close to a fence is affected in the case of a potential fence. Also, we observed that for fences with a weak restriction effect, the confidence interval of the fitted *L* increases, resulting in a less precise estimation of *L*. The fitted *D*_0_ are close to the value of 0.8 μm^2^ s^−1^ used in the probabilistic fence model. This shows that the additional forces from the potential fences do not change the diffusion of a single particle.

**Figure 7.**
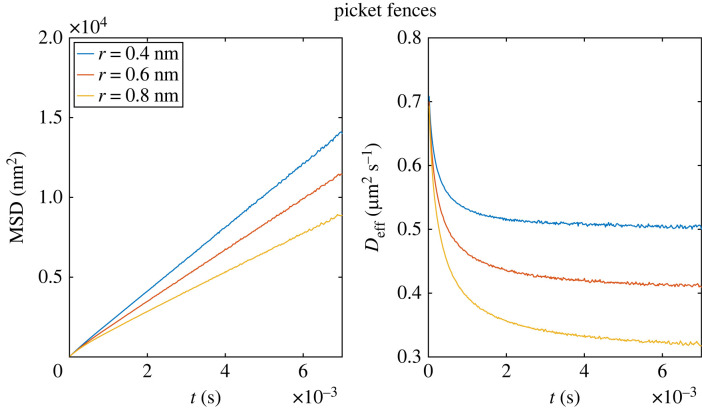
MSD and effective diffusion coefficient over time for a single particle with varying radius. All other parameters are the same as in §2.2.

The restricting effect of the picket fence (given the range of picket radii, free spaces between pickets, and the small particle radius) is small compared with the effects of the other two fence models and increases for smaller *l*_free_ or larger pickets. For a larger diffusing particle, the restriction effect of the picket fence increases, as shown in figure 7. For the probabilistic fence and the potential fence, the restricting effect is independent of the particle size (see electronic supplementary material, appendix).

### 3.5. Particle interaction

It is central for analysing reaction–diffusion dynamics to simulate multi-particle systems. To analyse the effect of the different fence models on the diffusion of multiple particles, we simulated the diffusion of a varying number of particles in a small, periodic region with (*a*) no fences, (*b*) probabilistic fences, (*c*) potential-based fences and (*d*) picket fences. The parameters are as described in §2.2 with a varying number of particles and the timesteps found in figure 5 (d*t* = 2 ns for the picket fences and d*t* = 20 ns for the other two fence models and the fence free simulation).

**Figure 8.**
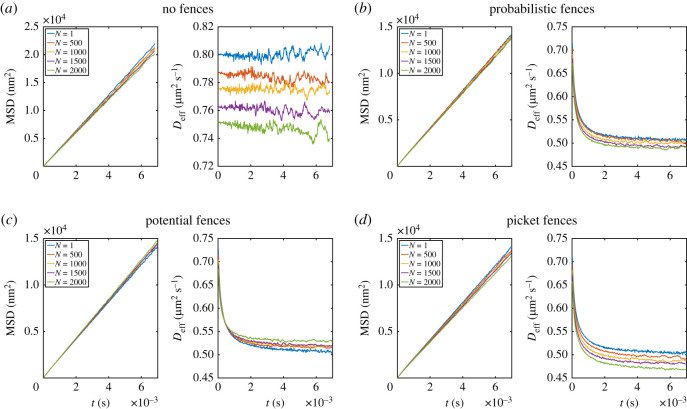
MSD and *D*_eff_ for a system with different numbers of particles (1, 500, 1000, 1500 and 2000) with (*a*) without fences, (*b*) probabilistic fences, (*c*) potential fences and (*d*) picket fences.

For the free diffusion, we found that the particle interaction slows down movement (figure 8*a*), suggesting a slower reaction speed according to the two-dimensional Smoluchowski rate [57] as well as a slower diffusion into the equilibrium of particles introduced locally. This effect is in line with results from several MD studies [33,44,58].

Similar to the free diffusion, we found a slowed macroscopic diffusion due to particle interactions for the probabilistic fences and the picket fences (figure 8*b*,*d*). In the case of the potential fence (figure 8*c*), we observed that the effective diffusion *D*_eff_ for large focal areas increases with the number of particles. This appears to be a bias introduced by the potential fences. The energy added to the system by the potential fences evaporates (due to the overdamped Langevin limit) in the single-particle case. However, when a particle moving from the fence collides with another particle (which is the more likely, the more crowded the environment becomes), this additional energy can take effect in the system leading to an increased diffusion.

### 3.6. Particle drift

**Figure 9.**
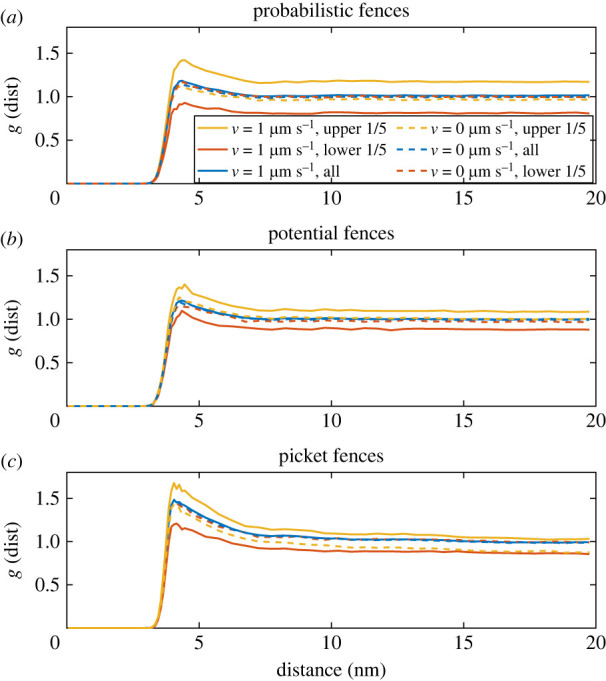
Radial distribution function *g*(dist) of 500 particles (*r* = 2 nm) that drift in the *y*-direction with a velocity of 1 μm s^−1^. Shown are *g*(dist) for all particles in the simulation regions and only for the particles in the upper and lower fifth of the simulation region.

Besides the random movement due to diffusion, a directed movement can be added to the particles. Here, we will test how the three fence models affect the dynamics if a drift is added to the particle movement. We use a larger radius of 2 nm for the drifting particles, since these experiments are often performed with proteins or microdomains in the membrane [10–12]. The free space between the pickets is enhanced to 2 nm, and *k*_fence_ and *P*_Hop_ are chosen to give similar macroscopic *D*_eff_ as the picket fence. The simulation region is enlarged to 500 nm in the *y*-direction with repulsive boundaries. In the *x*-direction, the region stays at 100 nm edge length with periodic boundary conditions. A more extended region in the drift direction combined with the repulsive boundaries allows us to study differences in the local particle density. If the simulated region is too small, the particle density might not change notably over the short distance. All particles drift in the *y*-direction with a velocity of 1 μm s^−1^, which is in the range of values reported for drifting proteins in model membranes [41,42]. Figure 9 shows the radial distribution function $g(\mathrm{dist})=1/\rho \sum_{i=1}^{N}C_{i}(\mathrm{dist}-dr/2,\mathrm{dist}+dr/2)/A_{i}(\mathrm{dist}-dr/2,\mathrm{dist}+dr/2)$. Here, *C*_*i*_(dist − d*r*/2, dist + d*r*/2) is the number of particles within a distance of dist − d*r*/2 to dist + d*r*/2 from a reference particle, *A*_*i*_(dist − d*r*/2, dist + d*r*/2) is the area observed around the reference particle, and *ρ* is the density (*N*/*A*) of the full simulation region. Consequently, *g*(dist) is the local density of particles around a reference particle at different distances. It is calculated for all particles in the system and those located in the upper and lower fifth of the simulation region averaged over the last 0.4 s of the 0.5 s long run. After the initial 0.1 s, the mean *y*-position of all particles has shifted from the centre of the region to their new, stable value. For all three fence models, the additional drift induces a higher local density in the drift direction. In the case of the probabilistic fences, this effect is most pronounced. For this fence model, it is possible that two particles are nearby but divided by a fence. In the case of the potential fences and the picket fences, excluded areas are created along the fences that reduce *g*(dist). These excluded regions make it less likely that particles from different fenced regions are interacting. Despite the location of the particles or the chosen fence model, *g*(dist) goes to zero for small distances. This shows that the excluded volume effect of the moving particles remains unaffected by the additional drift.

## 4. Discussion and conclusion

This study aimed to investigate three different ways to model actin fences in particle-based reaction–diffusion simulation: probabilistic fences, potential fences and picket fences. Thereby, our focus has been on the computational cost and artefacts introduced by the respective methods in single-particle and many-particle systems. We implemented our own simulator to realize and study all three fence models. For the diffusion, we used the overdamped Langevin propagator, as it is used as the major propagator in particle-based reaction–diffusion simulation tools [25–28,36].

The runtime performance between the three fence models differs in terms of steps per second per particle (§3.2) as well as the usable timestep (§3.3).

In our implementation, the picket fence model was the fastest to simulate. The probabilistic fences show the worst performance due to the complex calculation of line intersections needed (§3.2). The differences in the required timestep are considerable if the diffusion of a single particle is simulated, where the picket fence needs a timestep of 2 ns. By contrast, the probabilistic fences can have timesteps 1000 times larger (d*t* = 2 μs), and the potential fences can have timesteps up to 200 ns for our model parameters. For many-particles systems, the differences are minor, with a timestep of 2 ns for the picket model and a d*t* of 20 ns for the other two fence models. Note that these timesteps are only valid for our model parameters and have the purpose of comparing systems that are identical except for the fence model. Other systems with different parameters may need another timestep. Due to complex interactions in the system, it is hard to predict the appropriate timestep. Additionally, the timestep-dependency of the probabilistic fence model (${\tilde{P}}_{\mathrm{Hop}}=P_{\mathrm{Hop}}/\sqrt{dt}$) should be kept in mind. However, d*t* can be identified by testing. If the timestep is sufficiently small, the results may not change with the timestep. A few helpful rules of thumb exist to find an appropriate d*t*. (i) The displacement due to diffusion within a step scales linearly with the square-root of the diffusion coefficient and timestep. Consequently, a faster diffusion needs a smaller timestep. (ii) If the characteristic length *L* is reduced, which implies a higher number of fences in the simulated area, the timestep should also be reduced to ensure correct sampling of the interaction between particles and fences. (iii) The displacement due to interactions scales linearly with the force and timestep. Therefore, a larger interaction potential needs a smaller timestep.

Besides the computational cost, all three approaches are suitable to model single particle diffusion in structured membranes (§3.4), and thus applicable to support wet-laboratory experiments aimed at investigating the lateral diffusion of membrane-bound particles [30]. The potential fences need a slightly larger fenced region to produce the same restriction effect (see §§3.4 and 3.1), and for large hop probabilities/small energy barriers, the impact of *L* on the restriction decreases as shown by the large confidence interval in figure 6*b*.

We also investigated the effect of fences on differently dense populated systems. We observed a slowed diffusion for a fence-less system, which is in line with results from other studies [33,44,58]. Probabilistic and picket fences show the same trend, whereas the macroscopic diffusion coefficient in crowded environments increases slightly for potential fences. As the diffusion coefficient is one factor that determines the reaction speed [57], this indicates that the reaction speed would slightly differ between the methods.

The effect of the picket fences on diffusion depends on the particle size, while the impact of the probabilistic fence and the potential fence are independent of particle size. However, the role the particle size plays in the restricted movements in cellular membranes is not clear. Fujiwara *et al.* [19] reported the effective (macroscopic) diffusion coefficient of DOPE (lipid) and TfR (Transferrin receptor) in different cell lines. Whereas in cell lines, such as NRK, DOPE has a larger macroscopic diffusion coefficient than TfR, in some cell lines, such as PtK2, both particles show, despite TfR being larger than DOPE, the same macroscopic diffusion coefficient. According to the diffusion model by Hughes *et al.* [59,60] and wet-laboratory experiments [46], the smaller particles (DOPE) should diffuse faster than the bigger ones (TfR) at the microscopic scale. Consequently, to observe the same macroscopic behaviour for both particles, the movement of the DOPE molecule must experience additional restrictions in comparison with the bigger TfR molecule in these cell lines.

This indicates that the effects of fences on different molecules vary with the cell line. Additionally, other factors, such as microdomains with their higher viscosity [61], might influence the movement too. Therefore, to study the diffusion of different particle types, it would be necessary (and possible in each of the approaches) to attribute each particle type its own ${\tilde{P}}_{\mathrm{Hop}}$, *k*_fence_ or particle–picket interaction term.

In addition to pure diffusion, we investigated the effect of an additional drift compatible with all three fence models. Such a drift is observed for proteins or lipid rafts in cells stimulated by an external electric field [10–12]. We found that all fence models allow the creation of a concentration gradient due to the drift. Considering fences in models investigating the drift of particles would help make the system more realistic and understand the underlying mechanism of cells, such as reacting to external electric fields [7].

We have not addressed the issue of anomalous diffusion and different diffusion modes due to it being outside the scope of this work. Anomalous diffusion is characterized by the MSD being sublinear in time. However, it can be difficult to detect in experimental settings due to issues associated with ensemble and time averaging [62]. This is partly because an MSD approach is a blunt tool, and over initial times the measured MSD can be sublinear and then become linear at later times. Approaches such as single fluorescent molecule video imaging [63] and single-particle tracking (SPT; see [62] for a nice discussion) have been crucial in elucidating the dynamics of particle motion on the plasma membrane. But despite these developments, typical single-particle tracks do not have enough data points to perform MSD analysis effectively (see also discussion by Michalet [64,65]). Robson *et al.* [66] have developed a Bayesian inference approach that attempted to distinguish, with some success, tracks characterized by four diffusion models: Brownian, anomalous, confined and directed using total internal reflection fluorescence (TIRF) microscopy. Priors were formulated from both experiments and simulations. However, very recently, there has been a number of studies based on interferometric scattering microscopy (iSCAT) that overcomes the short trajectory issues associated with SPT. De Wit *et al.* [67] used iSCAT to track membrane proteins in live cultured mammalian cell lines with 3 nm precision and 25 μs temporal resolution. Taylor *et al.* [68] developed new imaging techniques that have overcome speckling issues associated with iSCAT, while Taylor & Sandoghdar [69] have given a review of iSCAT. Reina *et al.* [31] have also used iSCAT to explore lateral membrane diffusion dynamics and to characterize different diffusion modes along with average compartment size, location uncertainty and diffusion rates.

Lastly, we want to summarize our observations to help modellers to find the right approach. For single-particle systems, all three fence models can be used to restrict the diffusion of a particle. However, the picket fence needs a much smaller timestep than the other models, making it computationally costly. Also, one should be aware that the potential fences have a finite thickness and thereby affect the particle density in their proximity. By contrast, the probabilistic fences are infinitely thin, as shown in figure 2.

In the case of many-particle systems, the restriction of movement decreases with the number of particles for potential fences. In contrast to potential fences, probabilistic and picket fences show the expected behaviour for many-particle systems. It should be noted that the probabilistic fence model allows particles from different compartments to collide due to the infinitely thin fence. This influences the modelling of bimolecular reactions in a many-particle fenced system. For potential and picket fences, this is not the case. In conclusion, no superior method for all settings could be identified. However, we identified crucial aspects to be considered in concrete applications.

## Data Availability

The code of the simulator used in this study, data for plotting, and the scripts to plot the figures are available on OSF (https://doi.org/10.17605/OSF.IO/MHX7D) [38]. The data are provided in electronic supplementary material [70].
